# Use of animal biometrics for accurate hunting evidence of wild ungulates: red deer as a model species

**DOI:** 10.3389/fvets.2026.1736979

**Published:** 2026-02-24

**Authors:** Ondřej Kanich, Jan Cukor, Jana Adámková, Vlastimil Skoták, Martin Sakin, Veronika Olejníčková, Tomáš Volf, Martin Drahanský, Vlastimil Hart

**Affiliations:** 1 Department of Anthropology, Faculty of Science, Masaryk University, Brno, Czechia; 2 Department of Silviculture, Faculty of Forestry and Wood Sciences, Czech University of Life Sciences Prague, Prague, Czechia; 3 Department of Game Management, Forestry and Game Management Research Institute, Jíloviště, Czechia; 4 Department of Game Management and Wildlife Biology, Faculty of Forestry and Wood Sciences, Czech University of Life Sciences Prague, Prague, Czechia

**Keywords:** hunting statistics, LoFTR, muzzle pattern recognition, overabundance, wildlife management

## Abstract

Central Europe faces an overabundance of wild ungulates, which is driven by several factors, including traditional hunting practices. The harvest of females is insufficient and recorded without verification, even when they were not actually hunted. This practice contributes to further population growth through accurate hunting records. Therefore, basic procedures for automated registration based on muzzle pattern animal biometric evaluation of harvested wild ungulates were proposed. The red deer (*Cervus elaphus*) served as the model species. For the assessment of biometric characteristics, 2,193 photographs were taken from the frontal and overhead directions of 972 harvested red deer during regular game management. A comparison of the collected images using the LoFTR (*Local Feature TRansformer*) method revealed the potential for individual identification, with the peak accuracy of 95.048%. On the contrary, the minimum accuracy was 90.048% using a combination of overhead and frontal images of high and medium quality. Because there is no solution for the recognition of ungulates the comparison of these results was performed with the recognition systems for pets and livestock. Achieved accuracy is around 2% better than comparable recognition systems (with similar dataset size, number of feature points, etc.). The results confirmed that biometric methods can be used to identify and record harvested game. This can be achieved by developing a mobile application that transmits images for automated comparison and evaluation. Once individual identity is confirmed, the animal will be registered. This ensures a verifiable record of harvested game and provides a solid foundation for sustainable hunting planning.

## 1 Introduction

In recent decades, populations of wild ungulates have been steadily increasing across Europe (1–3) which led to challenges for sustainable wildlife management (4). With increasing populations of major ungulate species, both native and introduced, a wide range of negative impacts on ecosystems and the management of natural resources, such as forestry and agriculture, has been observed. Among the primary concerns associated with overabundance is the detrimental impact on natural forest regeneration by browsing, fraying damage, and bark stripping in older stands (5). In an agricultural landscape, the damage is associated with grazing, trampling, and rooting of crops, which can result in damages amounting to tens of millions of euros annually, as was evaluated in the Czech Republic (6). Based on the population increase and the related negative impacts, it is possible to point out the overabundance of ungulates according to biological, ecological, and socio-economic criteria at the local level in Europe (1).

Therefore, effective population control methods are sought. Standard hunting management still represents the crucial and most widespread solution, though in many cases, it is inefficient and puts more pressure on hunters (7). As the population of ungulates increases, so does the number of harvested individuals, highlighting need for reliable and verifiable systems to track these individuals and their hunting evidence (8, 9). Currently, the standard methods for recording game harvests rely heavily on self-reported data provided by hunters and local hunting organizations, in particular hunting districts (10). Although hunting records are mandatory in many countries, these records are often subject to errors, inconsistencies, or even deliberate misreporting (10), especially in cases where there is high pressure on local hunters to increase hunting bags, such as in the Czech Republic. This may result in reported amounts being higher than the actual number of individuals that were really hunted (especially females) (8, 11). Consequently, this creates a significant gap between reported and actual harvests, ultimately reducing the reliability of population models and limiting the ability of authorities to develop evidence-based management strategies.

To address this issue, there is a growing interest in applying animal biometric recognition methods for wildlife evidence, which could guarantee verifiable records of harvested wildlife individuals. In this context, the use of biometrics is proposed—a method that has already been validated in the past for the identification of livestock and domestic animals (12–16). Economically, reliable identification of livestock is essential for breed differentiation, official registration, traceability across production systems, veterinary care, and the prevention of false insurance claims. Following the success of biometrics on livestock, similar techniques in harvested wild ungulates, based on muzzle pattern (*planum nasolabiale*), may allow for the identification of individuals through features analogous to human fingerprint minutiae (17, 18). In recent years, with the growing interest in the application of computer vision and deep learning in the veterinary field (19–21), studies on animal biometrics based on muzzle recognition have increased. These studies often used machine learning (22) or deep learning techniques (23, 24) to track the muzzle prints of various livestock or domestic species, including horses (*Equus ferus caballus*) (25), pigs (*Sus scrofa domesticus*) (26, 27), and dogs (*Canis lupus familiaris*) (24, 28, 29). However, the majority of research focuses on cattle (*Bos taurus*) (13, 23, 30–43). In those species, the technique has been successfully applied to individual animal identification, health and welfare monitoring, and traceability within food production systems. Despite its success in livestock, muzzle-based biometric identification has not been applied to wild ungulates yet, and studies in free-ranging populations are absent. The acquisition of muzzle in wildlife introduces specific challenges, including field conditions, variation in tissue integrity post-mortem, and, of course, the willingness and commitment of the hunters themselves to carry out the recording (17, 23).

Consequently, this study seeks to assess the viability of using muzzle pattern characteristics as a biometric identification tool for harvested ungulates’ evidence. This approach could facilitate effective hunting management following verification and potential practical implementation. Therefore, the primary study aim is to evaluate the proposed method on the red deer (*Cervus elaphus*), as this ungulate species is common across Europe. The particular objectives are to: (i) assess the biometric properties (mainly uniqueness, measurability, and performance) of muzzle pattern biometric characteristics; (ii) evaluate the practical usability of acquisition under field conditions; and (iii) evaluate the image of an individual’s muzzle pattern with the rest of the database to evaluate the precision of the proposed methodology. By adapting modern machine learning methods, we aim to bridge the gap between traditional wildlife management practices and modern digital tools, thereby contributing to more transparent and scientifically grounded hunting management systems.

## 2 Materials and methods

An animal biometric recognition system for wild ungulates (ABRSWU—Animal Biometric Recognition System for Wild Ungulates) was developed, consisting of several interdependent components, similar to other biometric systems. The first component is the capture device, responsible for acquiring images of the muzzle surface and other relevant data for the particular wild ungulate species. This data is then used to build the database, which represents the second step of the system. The core components of the solution include preprocessing, feature extraction, and comparison methods. All biometric terminology used in this study follows the definitions of the biometric vocabulary standard (44).

### 2.1 Data acquisition

**Figure 1:**
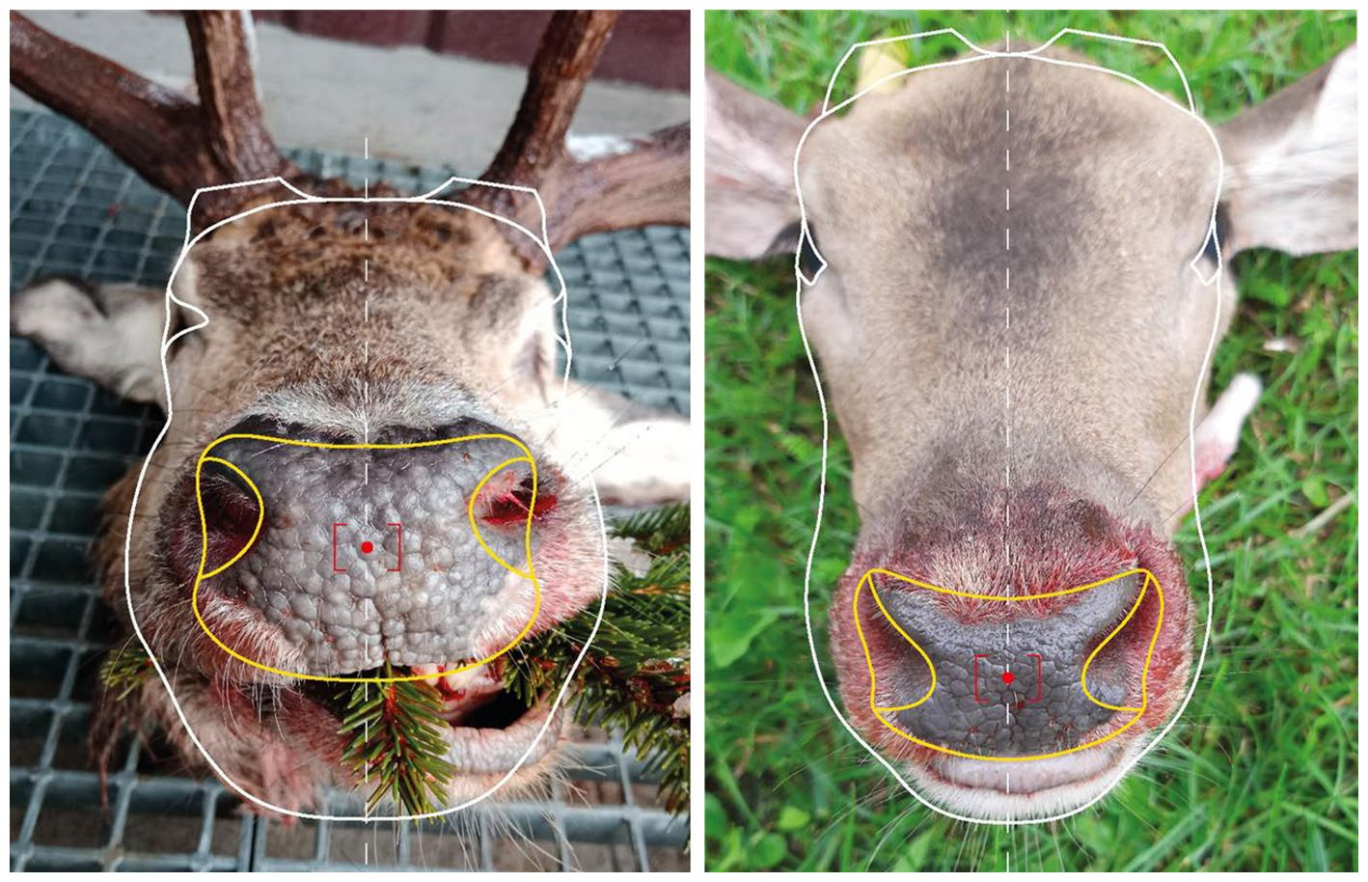
Example of the frontal (left) and overhead (right) images captured by the application with muzzle outlined in yellow.

The biometric comparison methods primarily rely on machine learning techniques. The success of these neural networks is crucially dependent on the datasets used, vis-à-vis the quantity and quality of the data used, such as a photo of the muzzle surface in this case. Only then can the database be transformed into a perfectly functional dataset. Therefore, due to the potential for using animal biometric comparison in wildlife management practice, the photos of muzzle were collected by cameras on a standard mobile phone. The evaluation was divided into two phases. The first one took place from November 2023 to August 2024 and contained common wild ungulate species (see Table 1). The second phase followed the first one, and it lasted until August 2025. It used a specialized application (further information is given later—see Figure 1) and was focused on red deer only.

**Table 1:** List of animal species recorded during the first phase.

English name	Latin name	Individuals	Photos
Fallow deer	*Dama dama*	391	1,042
Red deer	*Cervus elaphus*	338	924
Sika deer	*Cervus nippon*	244	592
Roe deer	*Capreolus capreolus*	1,268	2,896
European mouflon	*Ovis aries musimon*	216	559
Total		2,457	6,013

In the first phase, hunters captured images of the muzzle surfaces of wild ungulates at a minimum Full HD (1,920 × 1,080 pixels) resolution, using a wide range of their mobile phones. Because the main aim is to propose and validate a simple field method, the hunters who took photographs of harvested game received these basic instructions for mobile-phone camera settings as follows: digital zoom and macro mode were disabled. Furthermore, the muzzle had to be cleaned from the blood, mud, or other dirt, and the camera was required to focus precisely on the surface structure. At least two images were taken from every hunted individual. First, a frontal image was taken from a distance of 6–15 cm, ensuring that the entire head was visible and, in males, at least part of the antlers was captured for basic categorization of the sex of harvested individuals. Second, an overhead image was taken from approximately a 45° angle above the head, focusing on the muzzle.

All photographs were collected in hunting districts across the entire territory of the Czech Republic, ensuring nationwide geographical coverage for all ungulate species included in the dataset. This way, 2,457 individuals predominantly from the family Cervidae, with 6,013 photos, were acquired (for exact values see Table 1) and used in the first phase of testing (see Results).

During the second phase, a mobile application was developed for data acquisition, specifically for photographing the muzzle pattern. The main advantage was the implementation of a graphical overlay. This was created to follow the morphology of the Cervidae head. This mask is displayed when the camera is activated, allowing the background outside the outlined area to be faded out. Separate overlays were designed for frontal and overhead photographs. The application also includes a form for entering information about the hunted individual and the hunter. In addition, it automatically records metadata related to each image. The use of the application for capturing suitable photographs is illustrated in Figure 1. The application can automatically send the data into the database or save the complete package, which can be sent later (for example, when the connection is better). The second phase of testing focused on red deer; altogether with the first phase, 972 individuals were acquired (with 2,193 photographs).

In the second step of database acquisition, each photograph was manually inspected and annotated. For annotation, the LabelMe software was used. A rectangle was used to delineate the muzzle, two circles marked the positions of the nostrils, and two additional rectangles indicated the locations of the antlers (when present). The basic classification of image quality of photos submitted by hunters from routine hunting practice was done manually, based on general visual criteria. Three quality levels were assigned to the images. High-quality photographs were defined by proper lighting, clear contrast of the muzzle surface, sharp focus, and correct positioning. Medium-quality images were acceptable, with clearly visible and identifiable muzzle structure. The lowest quality category included unusable photographs—those lacking the animal altogether, showing the entire animal instead of the muzzle, focused on the hunter or background, or where the muzzle pattern was obscured, unreadable, overexposed, underexposed, blurred, or incorrectly positioned.

### 2.2 Animal biometric recognition of muzzle pattern

The following comparison methods were evaluated: DenseNet combined with FAISS (Facebook AI Similarity Search), SIFT (Scale-Invariant Feature Transform), SURF (Speeded-Up Robust Features), ORB (Oriented FAST and Rotated BRIEF), HOG (Histogram of Oriented Gradients), Siamese networks, and handcrafted features analogous to fingerprint minutiae. All of these methods were tested during the first phase of the study.

DenseNet + FAISS combines a convolutional neural network—specifically DenseNet, in which each layer is fully connected to all preceding layers (45)—with the FAISS library developed by Meta, designed for efficient large-scale similarity search (46). The SIFT is a classical, non-neural algorithm used to detect key points in an image based on differences of Gaussian and to define descriptors for subsequent comparison (47). The SURF represents an accelerated version of SIFT, employing box filters instead of Gaussian differences and relying on the Hessian matrix for key point detection (47). The ORB is another classical approach that utilizes the FAST (Features from Accelerated Segment Test) algorithm to locate key points, and the BRIEF (Binary Robust Independent Elementary Features) descriptor to extract robust and computationally efficient image features (47).

**Figure 2:**
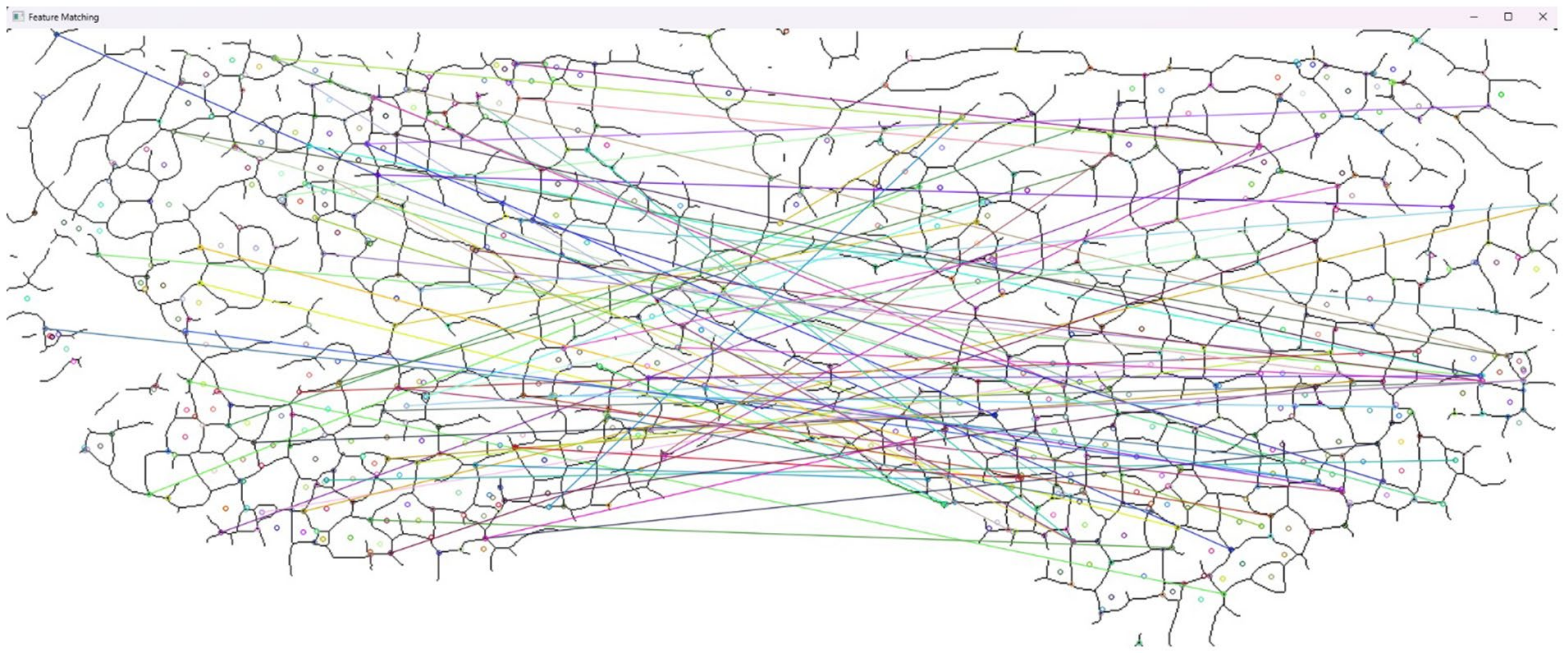
Example of muzzle pattern structure valley comparison.

The HOG is a descriptor for image data, dividing the image into cells and computing the histogram of gradients for each (also a classical algorithm) (48). A Siamese network is usually used with two neural networks containing the same architecture and shared weights; each network is given an image, and over time, it trains itself to measure the similarities between them (49). The method uses handcrafted features to compare the bifurcation of the valleys on the muzzle (similar to a fingerprint comparison). A graphical representation of the comparison of extracted valleys can be found in Figure 2. This method also uses only a classical approach.

**Figure 3:**
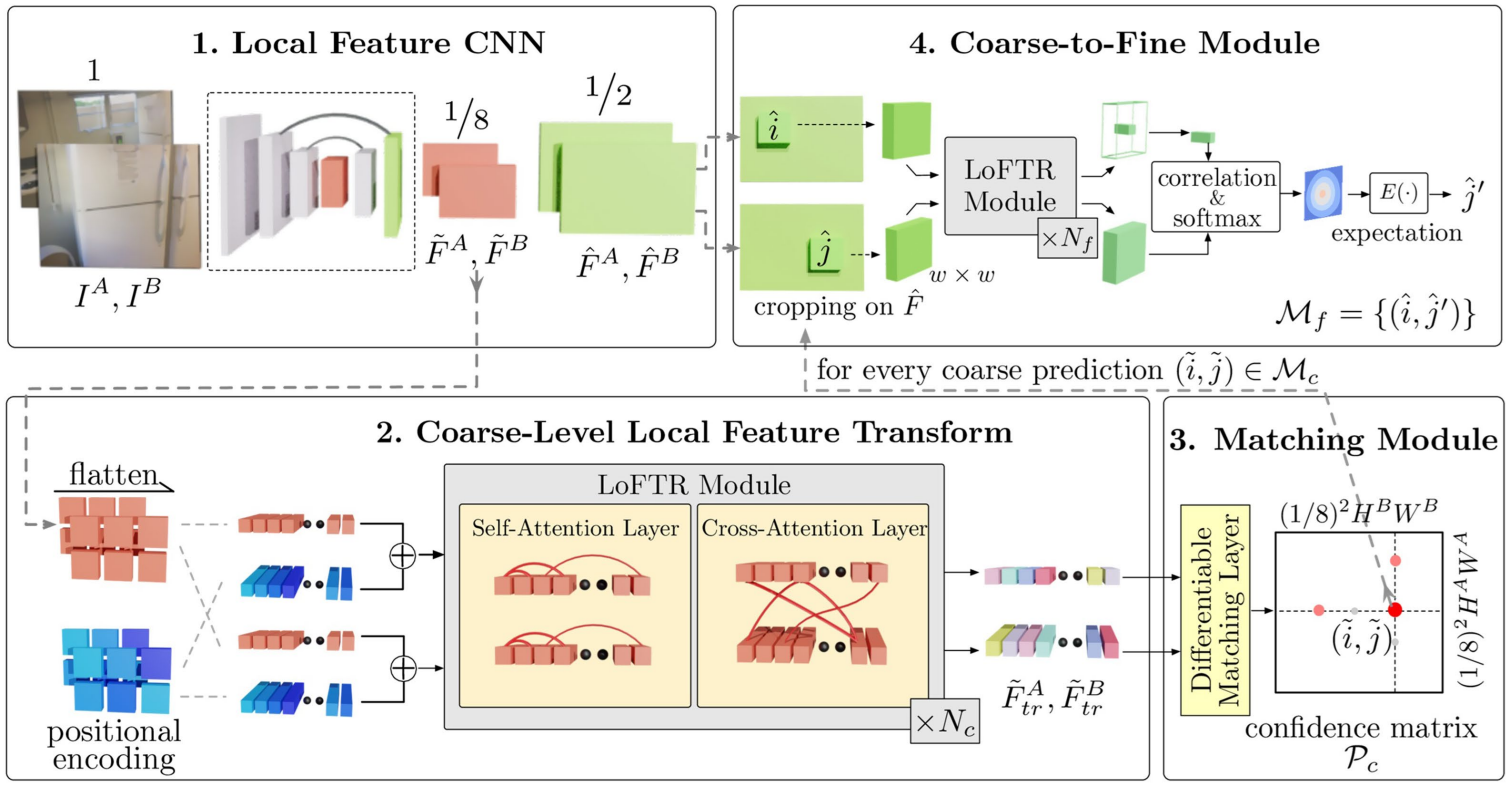
Overview of the LoFTR method, divided into four s (50). The first CNN section where coarse (red) and fine (green) feature maps are extracted. The second part involves flattening the coarse features and combining them with positional encoding. Afterwards, they are processed by a transformer encoder. The third part is where correspondences from the encoder are found, and the fourth is where the results are refined using fine features.

For the second phase of testing, the LoFTR (*Local Feature TRansformer*) matcher was chosen as the comparison method. All the information about the LoFTR method is taken from (50). The LoFTR is a deep learning algorithm using the Transformer architecture. It finds corresponding points between two images (detector-free) and then returns the similarity score. An overview of the method is shown in Figure 3.

**Figure 4:**
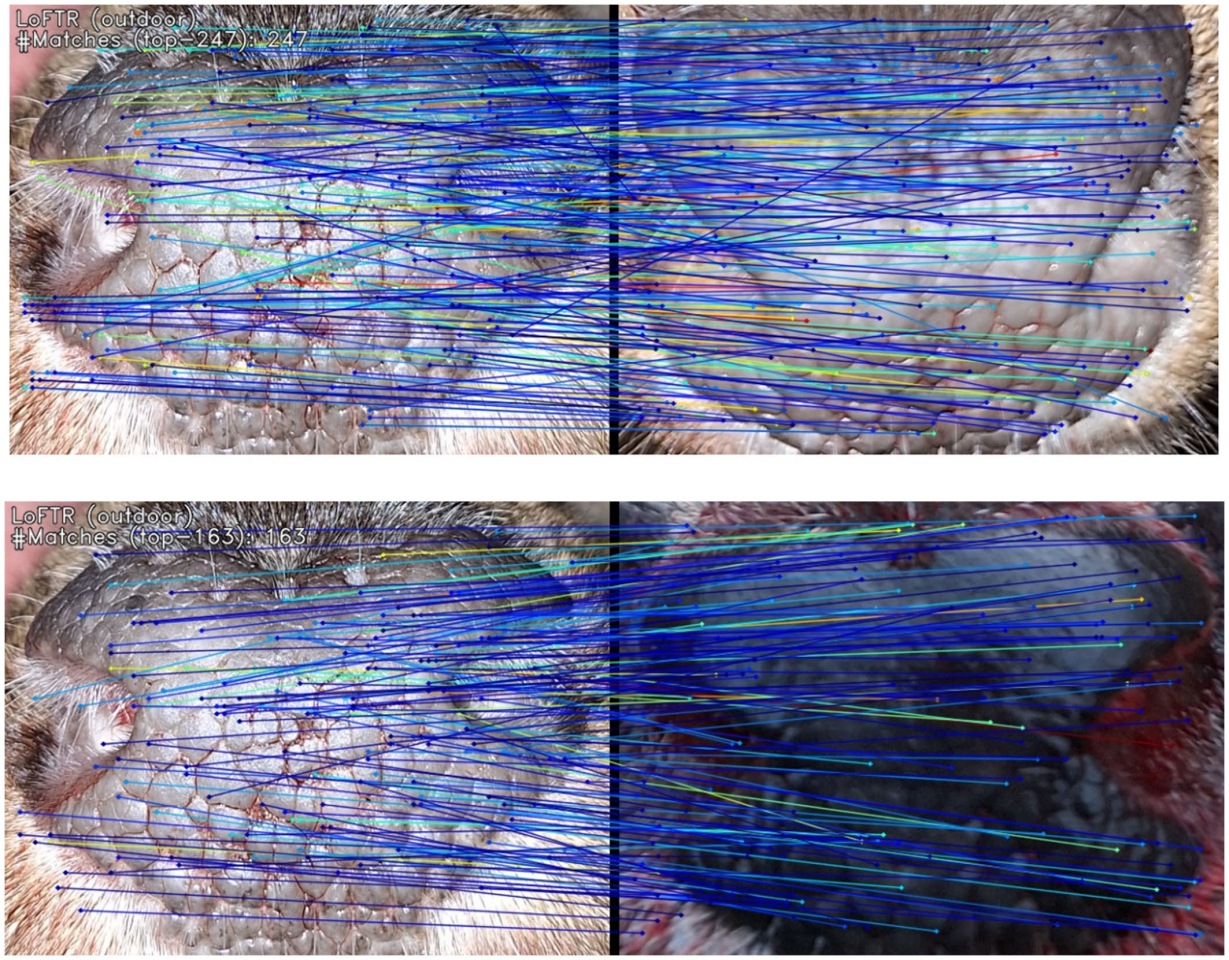
Visualization of verification using the LoFTR method. Blue and green lines connect areas where the corresponding key points in both images were found. The top picture shows confirmed verification with 247 corresponding key points found. The bottom image shows denied verification with only 163 corresponding key points found.

The first part uses a local feature CNN (*Convolutional Neural Network*), which extracts feature maps (coarse–red, and fine–green). The coarse features are flattened into a one-dimensional vector and put together with positional encoding for the second part. These are processed by another neural network (specifically, a transformer encoder, using self-attention and cross-attention layers). In the third part, correspondences between transformed coarse-level features are found (using a differentiable matching layer). Each coarse-level prediction is refined using a local window and fine-level features. The whole technique was pretrained on several million images (indoor and outdoor). In this case, the pretrained *outdoor-ds* checkpoint was employed in this study. Graphically, the comparison can be visualized as shown in Figure 4. The image on the top shows confirmed verification (visualizing 247 corresponding key points), the image below shows denied verification (visualizing 163 corresponding key points).

**Figure 5:**
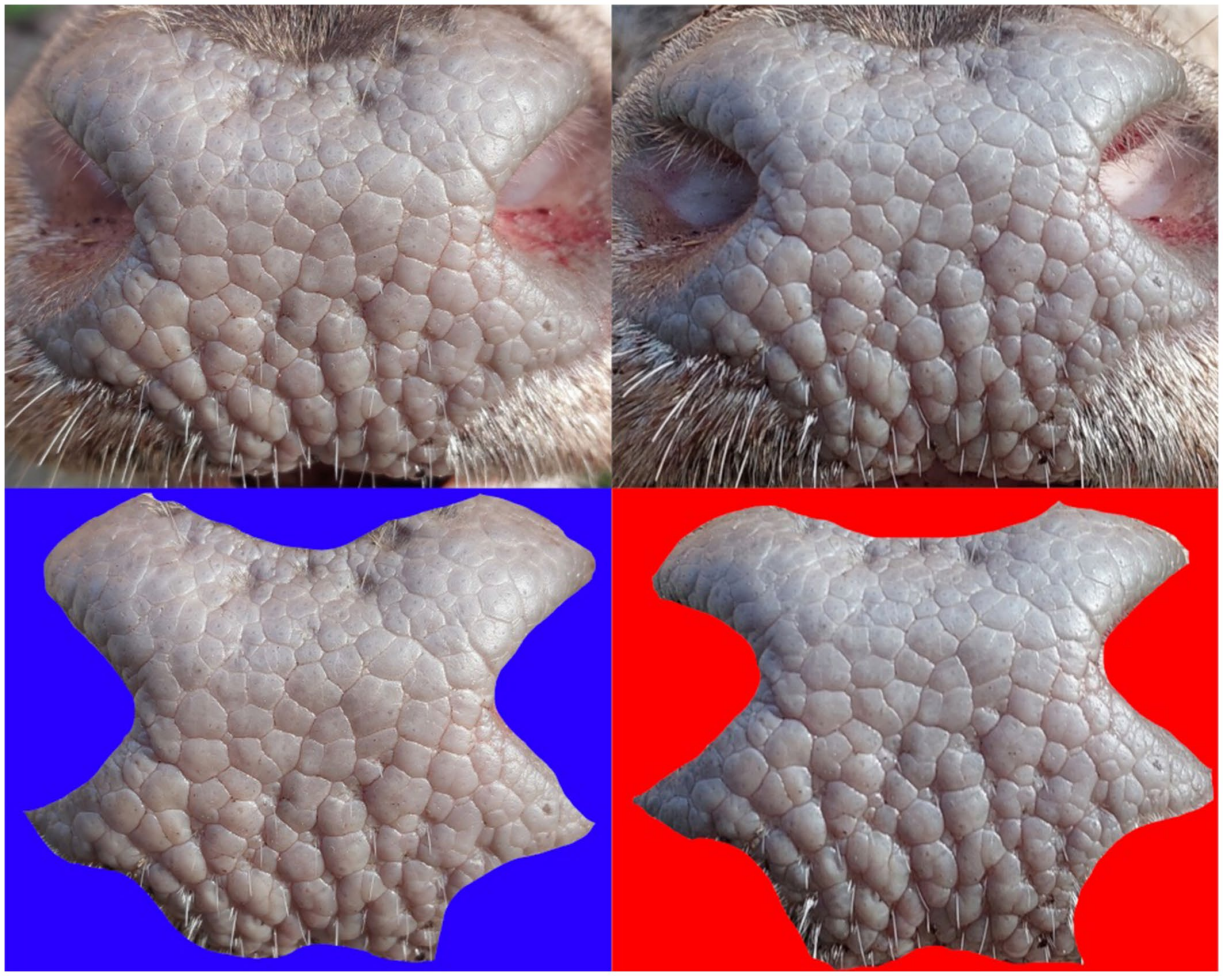
Examples of cropped images used for training and testing prior to preprocessing steps. The top images display the muzzle structure without special trimming, while the bottom images show the muzzle with special trimming.

For comparison of individuality, only the muzzle was used (otherwise, there could be a lower accuracy due to finding similarities in buildings and nature in the background). More specifically, a rectangle around the muzzle was used, as evident from Figure 4. Some key points were outside of the muzzle (e.g., on hair). Therefore, the effect of precise trimming of the muzzle area was tested. An example is shown in Figure 5, where the similarity score in the first row is 70 and 60% in the second. Using a rectangular region of interest proved to be both faster and more accurate; hence, additional trimming was not required. Nevertheless, several basic preprocessing steps were applied before using the LoFTR method. Specifically, each image was resized to 480 × 640 pixels, converted to greyscale (single color channel), and normalized to a mean value of 0.485 and a standard deviation of 0.229.

### 2.3 Dataset creation and training process for LoFTR

Before presenting the exact results, it is essential to describe the training and testing procedure, which consisted of two main components: *dataset creation* and *threshold estimation*. The first step in creating a the dataset involved dividing the database according to image quality and capture angle—specifically into high-quality, medium-quality, frontal (0°), and overhead (45°) categories. Based on these criteria, four datasets were established: (1) high-quality frontal (0°) images; (2) combined high- and medium-quality frontal (0°) images; (3) high-quality frontal (0°) and overhead (45°) images; and (4) combined high- and medium-quality frontal (0°) and overhead (45°) images.

For each category, training and testing sets composed of image pairs were generated, with an equal split—50% of the data used for training and 50% for testing and evaluation, with no crossover. Because the database reflected real-world conditions, which resulted in a substantial imbalance existed between the number of images representing the same individual and those of different individuals (i.e., many images of different animals, but only a few per specific individual). Within each set, pairs of animal photographs were generated and labeled according to whether they depicted the same or different individuals. The generation process maintained a predefined ratio between matching (same individual) and non-matching (different individual) pairs, which in the present red deer experiments ranged from 0.1 to 0.5.

The training set was used to determine the optimal threshold for distinguishing whether a pair of images represented the same individual. This is accomplished by evaluating the entire training dataset and selecting the lowest possible threshold value that produced no false positives. The identified threshold was then applied during classifier evaluation on the testing dataset. Classifier outputs were subsequently compared with the ground truth for each image pair, and performance metrics such as accuracy, precision, and false positive rate were calculated based on these comparisons.

### 2.4 Data analysis

Once the essential parts were prepared (neural networks trained, threshold defined, etc.), their accuracy was computed. Information about metrics was taken from ISO/IEC 19795-1:2021 (51) in this section. Pairs of images from a particular dataset were fed into the algorithms, and the results was comparison score. If the comparison score was higher than the threshold, the images were declared to be from the same individual. If the computed score was lower, the images would be from a different individual. This automated answer was then compared with the ground truth. After that, the performance statistics could be computed.

All the metrics were computed based on the following definitions: *true positive* (the number of the same individual image pairs identified as such), *true negative* (the number of different individual image pairs identified as such), *false positive* (the number of the same individual image pairs incorrectly identified as different—also referred to as type I statistical error) and *false negative* (the number of different individual image pairs incorrectly identified as the same—also referred to as type II statistical error).

*Accuracy* is defined as the sum of true positives and true negatives divided by the total number of image pairs in the dataset (52). The *True Match Rate* (TMR) is the number of true positives divided by the number of the same individual image pairs. The *True Non-Match Rate* (TNMR) is the sum of true negatives divided by the number of different individual image pairs. The *False Match Rate* (FMR) is the number of false positives divided by the number of the same individual image pairs. The *False Non-Match Rate* (FNMR) is the sum of false negatives divided by the number of different individual image pairs. In machine learning, accuracy values are usually compared when evaluating different methods. However, for practical applications, FMR and FNMR (or TMR/TNMR) are generally more understandable.

The evaluation results of the LoFTR method (*accuracy*) were statistically compared between selected categories using comparison of multiple binomial proportions (53). Confidence interval limits for each variant were computed using beta-distribution. The results of this analysis were visualized by bar plot with error bars. This analysis was performed in R software (54), plot was created in its package ggplot2 (55). Alpha level of 0.05 was selected for statistical computations.

## 3 Results

The evaluation was conducted in two phases, as described in the Data Acquisition section. The first phase, carried out primarily in September 2024, utilized a multispecies database (see Table 1 for details). The dataset was used without additional filtering, except for neural network-based approaches, where it was divided into two equal parts for training and testing. The resulting accuracy values for the evaluated methods are presented in Table 2 (two best values in bold).

**Table 2:** Evaluation of the various methods on the first phase dataset.

Evaluation method	DenseNet + FAISS	SIFT	SURF	ORB	HOG	Siamese network	Handcrafted features
Accuracy	62.6%	60.1%	63.5%	**68.6%**	57.9%	65.3%	**67.4%**

Two main issues were identified: first, the overall performance of the tested methods remained below 70%; and second, as the database expanded, several initially promising approaches exhibited poor scalability. Since performance on large datasets represented one of the key evaluation criteria, the second phase was scheduled for the following year, focusing exclusively on a single species (red deer) and employing methods known to scale effectively with database size, specifically the LoFTR algorithm. The second phase of the evaluation pursued several objectives. The primary goal was to assess the accuracy of the selected method. The second objective was to examine the influence of *image quality* (high and medium) and *capture angle* (frontal and overhead) on performance. Finally, the third objective was to evaluate the effect of varying the ratio of generated pairs used for training and testing. The main performance metrics are summarized in Table 3.

**Table 3:** Evaluation of the LoFTR method on red deer datasets in the second phase.

Category	Accuracy	TMR	TNMR	FMR	FNMR
High, 0°	94.974%	92.063%	97.884%	2.116%	7.937%
High and medium, 0°	**95.085%**	**92.308%**	97.863%	2.137%	**7.692%**
High, 0° and 45°	93.154%	87.759%	98.548%	1.452%	12.241%
High and medium, 0° and 45°	90.048%	80.096%	**100%**	**0.000%**	19.904%

Table 3 shows that the best results (in bold) were achieved when high- and medium-quality images were used, but only the frontal (0°) ones. Using overhead (45°) images performed noticeably worse. The difference between frontal high and frontal high and medium was only marginal. The answer to the question of why the usage of lower-quality images was better than higher-quality images is presented in Table 4. The second category used 12.5% more images than the first.

**Table 4:** Images in datasets used in the second phase red deer evaluation.

Category	Pairs	Animals training	Images training	Animals testing	Images testing
High, 0°	378	411	583	390	550
High and medium, 0°	468	452	656	453	659
High, 0° and 45°	964	444	853	403	790
High and medium, 0° and 45°	1,246	463	967	478	989

**Figure 6:**
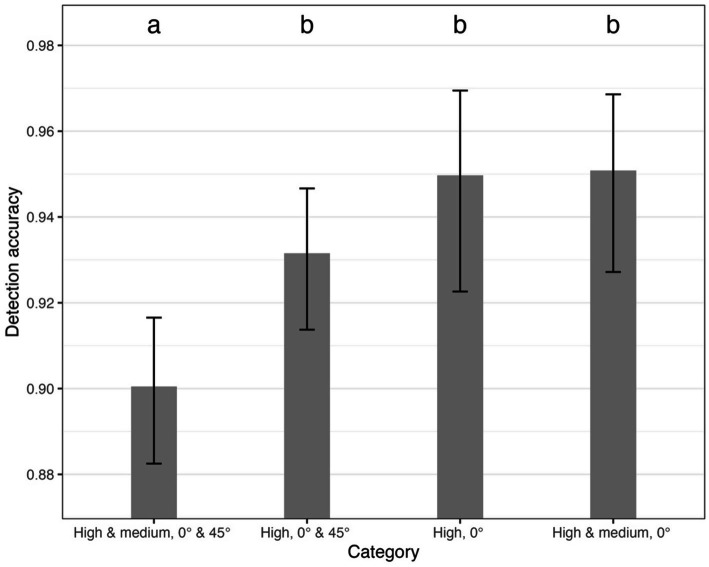
Comparison of model matching accuracy between selected variants. Error bars depict 95% confidence interval computed using beta-distribution. Indices above each bar depict statistical homogeneity (variants with identical index are not statistically different and vice versa).

A statistical comparison (Figure 6) of *accuracy* between the selected categories revealed a statistically significant difference for the category High and medium, 0° and 45° when compared with all other variants. This category exhibited an accuracy of 90.05%, whereas the accuracies of the remaining variants ranged from 93.15 to 95.09%. *p*-values and the studentized range (q) values (test statistics) for comparison of category High and medium, 0° and 45° to others were following: to High, 0° and 45° – *q* = 3.69, *p* = 0.04, to High, 0° – *q* = 4.71, *p* = 0.005 and to High and medium, 0° – *q* = 5.24, *p* = 0.001.

**Figure 7:**
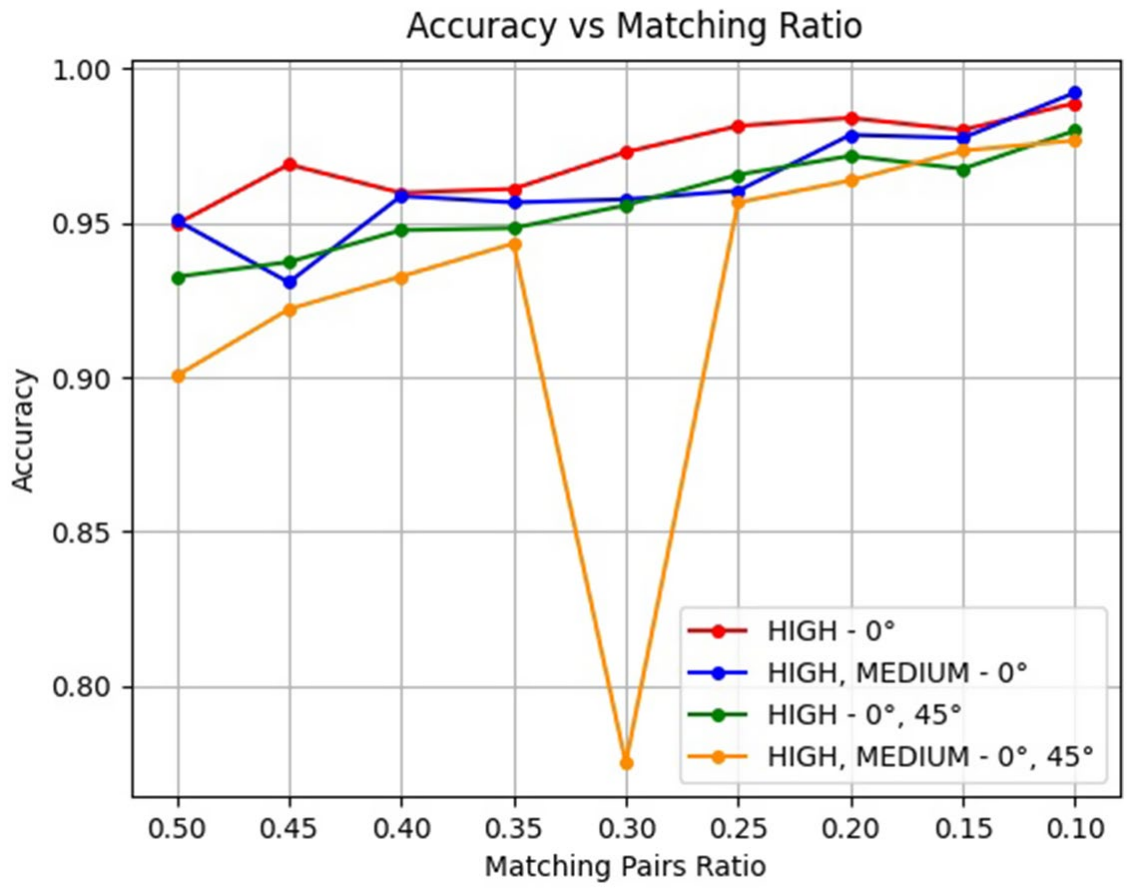
The relationship between the accuracy evaluation in testing and the ratio of genuine matching pairs used during the training phase is presented as percentages in decimal form. The red, blue, green, and yellow lines represent individual categories: high 0°, high and medium 0°, high 0° and 45°, and high and medium 0° and 45°, respectively. All graphs converge toward nearly perfect accuracy when the ratio of genuine matching pairs is low.

All of previous results were obtained using a 0.5 ratio between genuine and imposter pairs. The rationale for employing this balanced 50:50 ratio is illustrated in Figure 7. As shown, when the dataset becomes more imbalanced, the results appear artificially improved. This occurs because, at a 0.1 ratio, a method that classifies all images as different would still be correct in 90% of cases purely by chance. To avoid this statistical bias, a balanced 0.5 ratio was adopted. Additional insights from Figure 7 indicate that the frontal high-quality category seems to perform slightly better than the combined frontal high- and medium-quality category. Moreover, the results confirm that incorporating overhead (45°) images does not improve performance for this method. Based on the annotation, 95 images were of the lowest quality (unusable). This value could be utilized for the FTA (Fail to Acquire Rate), which would be 4.324%. Although the images were successfully captured, those deemed unusable were classified as “fail to acquire” rather than “fail to extract” cases [for a description of these metrics, see (51)]. Another limitation identified during the comparison process involved errors in database acquisition. Specifically, four animals produced highly similar images, and one case included photographs of two different individuals. These entries were subsequently blacklisted and excluded from the final evaluation.

## 4 Discussion

Given the specificity of the topic, which focuses on assessing the individuality of hunter ungulates, it is practically impossible to compare the obtained results with similar systems. Therefore, a comparison with livestock and pet records is a feasible alternative. In the case of cattle, Kumar et al. (23) propose a deep learning methodology across different identification scenarios with multiple test galleries, using 500 animals with 10 images each (5,000 images). The best result is achieved with *Deep Belief Network* (DBN), using 400 feature sets, with an impressive accuracy of up to 98.99%. When compared to the proposed ABRSWU solution, it should be noted that red deer muzzle surface is approximately half the size of cattle. This is important because the ABRSWU solution uses around 200 key points. In the study by Kumar et al. (23), the usage of 200 feature sets would lead to only 77.94% accuracy. The ABRSWU achieved 95% accuracy, and the dataset contained almost double the number of individuals (972 versus 500). Moreover, their study already proposed an application on the Android platform for the future.

Another study by Shojaeipour et al. (40) achieved similarly remarkable biometric identification accuracy of 99.1%. However, their research included fewer beef cattle animals (300 animals and 2,900 images) than our research. They used a tuned ResNet-50 model to extract muzzle features and ensure individual identification. The test-to-train set ratio was 80:20 in their case (compared to 50:50 in this study), which further improved the accuracy. Due to the larger area of the muzzle, they used images with a resolution of 1,024 × 1,024 (compared to 480 × 640). According to that study, using a similar resolution of 608 × 832, the accuracy reached 92.75% which is 2% lower than the ABRSWU method. Surprisingly, when compared to our study, almost double the images (268 images or 9.24%) did not capture the muzzle or were extremely blurry.

In the study by Nishanov et al. (43) 4,923 images of 268 cattle were used, along with 8 models for training. The DenseNet-121, WideResNet-50, and InceptionV3 models reached the highest accuracy rates of 99.2, 99.1, and 99.1%, respectively. This exemplifies the considerable impact that the number of animals in the database has. Using only a third of the individuals, the ABRSWU had an enormous impact on the results, as demonstrated by the first phase of this study. That is further enhanced by using more images. Deep learning employs fewer individuals and more images to better recognize the features of each individual.

A similar study also attempted to identify the nose pattern of dogs (28). Their research involved training with 1,035 images of 345 dogs, while testing was conducted on 828 images of 276 dogs. The highest accuracy recorded (in the top 1 results) was 92.39%. This was accomplished using a complex network of neural architectures and preprocessing techniques to generate a feature vector comparable to the minutiae of human fingerprints. In contrast, our study’s method utilizes a larger sample size leading to 3% improvement in accuracy despite employing only half the number of images. Compared to most of the studies on domestic animals mentioned above, our dataset is characterized by a significantly larger number of individuals combined with a relatively small number of images per individual. This makes the identification task considerably more challenging and the achieved performance particularly informative, as deep learning-based identification systems usually benefit from the opposite setting.

The determined accuracy of individual identification, which exceeded 95% for frontal photographs, provides a promising starting point for further method development and represents significant potential for practical application. If the best possible method were applied to the annual harvest of red deer in the Czech Republic, which amounted to 35,001 animals during the 2024/2025 hunting season (56), it would mean that 34,253 individuals could be identified as unique, while 748 would require confirmation by an alternative method. In this hypothetical scenario, there would be no cheating (i.e., the same individual seen as a different one). However, the evidence can be further refined in the future. The necessary steps are quality control and feedback when the image is acquired. After evaluation, it was also concluded that more focus should be placed on the quality of frontal images.

In addition to the quality of the acquired photo, the applicability of animal biometric identification method based on muzzle pattern could be affected by dermatologic conditions involving the analyzed area, similar to human fingerprints (57–59). Severe hand dermatitis has been shown to significantly impair fingerprint verification, with failure reported in 27% of patients with clinical dermatitis involving a palmar distal phalanx of either thumb (58). Other dermatologic conditions, such as psoriasis, eczema, and sclerodermatous diseases, have also been associated with disruption of epidermal ridge patterns, leading to reduced fingerprint recognition accuracy (59, 60). A comparable issue may occur in hunted ungulates. Papillomavirus infections lead to multiple benign fibropapilloma tumors, most frequently localized on the head, neck, abdomen, and extremities (61). Given the increasing prevalence of these infections (62), the presence of tumors on the muzzle may interfere with pattern recognition. Similarly, the other condition affecting the skin of the hunted ungulates, such as infection diseases (63–66), metabolic diseases, specifically zinc deficiency (67, 68) or plant-induced photosensitivity (69) can reduce the reliability of muzzle pattern characteristics. Although autoimmune diseases are less frequently reported in hunted ungulates than in domestic animals, it could potentially impair their muzzle surface (70). Additionally, skin neoplastic processes, particularly squamous cell carcinoma (SCC), are well documented in cattle (71) and have also been observed in wild ungulates (72). As SCC predominantly develops in areas exposed to sunlight, the muzzle is a common site of occurrence (73). Even though skin disease manifesting at the muzzle could interfere with the muzzle pattern-based identification, in the context of animal biometric identification applied post-mortem in hunted ungulates, images are acquired at a single time point. As a result, progressive skin disease or dermatologic conditions that do not affect a sufficient portion of the muzzle surface may have limited or no impact on the utility of muzzle pattern-based hunted ungulates evidence. Also, if the skin disease or dermatological condition change the muzzle pattern in a unique way, it could make the identification task simple and more reliable (similarly to tattoos or scars that make humans easily distinguishable).

Although the present study does not aim to address the animal welfare of harvested ungulates directly, it is worth noting that, if implemented in practice, this biometric identification method could indirectly contribute to the welfare and health status of wild ungulate populations. An overabundance of ungulates does not manifest only in negative impacts on the ecosystem and human land-use but also affects the condition of individuals of the given species (1). At sites with ungulate overabundance, a reduction in body mass can also be expected, according to the density-dependent concept in population dynamics (74). Moreover, for roe deer, intra-population stress could be expected in localities with high population densities due to males’ territorial behavior (75). In this context, the spreading of diseases is another aspect that could affect the health status of wild ungulate populations, including the most serious ones, such as African swine fever in the case of wild boars (76, 77). Nevertheless, the spread of disease can be mitigated through systematic population reduction, as regulated by the proposed control mechanism. For practical implementation, the final system should incorporate additional functionality. A key requirement is the inclusion of user authentication, enabling hunters and administrative personnel to log into the application. Furthermore, a comprehensive and clearly visualized database, accompanied by statistical summaries, is essential for reliable record-keeping. Whenever possible, data should be recorded automatically—particularly the date, time, and location (GPS coordinates) of each harvest—to facilitate accurate and efficient documentation via mobile devices. These parameters represent fundamental components internationally recognized as essential for reliable harvest records (8, 10). Automated data collection reduces errors and saves hunters’ time, a prerequisite for the system to become acceptable in everyday practice (13, 14). Additional key information that should be entered by the user includes the sex and age class of the harvested individual. For management purposes, it is also highly valuable to record the method of harvest. Individual or collective hunting or trapping can significantly influence population density, as well as the age and sex structure of harvested game (4, 7). Such data can then be used to recommend applying particular methods in other areas. Supplementary information, such as body weight, trophy parameters, or health status, can significantly enhance the scope of evaluation and is recommended for comprehensive population monitoring (11, 33).

The application should also operate on the principle of multi-level data access. This would enable hunters to produce a quick and simple record, which—owing to biometric traceability—is unambiguous and difficult to contest (17). Game managers or administrative authorities would then gain access to statistics and visualizations. These outputs should include harvest summaries by species, sex, age class, temporal trends, and spatial maps with options for comparisons across seasons or hunting grounds (1, 9).

An application designed this way creates a robust and transparent database based on biometric reliability and the automated collection of key information. This database can be used not only to fulfil legislative requirements, but above all, as a strategic tool for game management, enabling flexible responses to population dynamics, reducing discrepancies between planned and actual harvests, and mitigating the negative impacts of overabundant game on forest and agricultural ecosystems (1, 4, 5, 7).

## 5 Conclusion

The proposed method for identifying ungulates through the biometric features of the harvested individuals’ muzzle patterns demonstrates significant potential. Further development and refinement of the method are essential, as is the possibility of digitalizing and simplifying the traditionally conservative field of wildlife management. Based on the findings presented above, several conclusions can be drawn regarding the biometric properties of the ABRSWU system. All of the examined samples contained distinguishable muzzle pattern structures, indicating high *universality*. No identical or nearly identical muzzle patterns were observed, confirming a high degree of *uniqueness*. *Permanence* and *acceptability* do not present concerns in this context, as the images were obtained post-mortem. There were some issues with the quality of the images; nevertheless, no cases were found where the muzzle pattern could not be captured, i.e., *measurability* is high. *Price* and *maintenance* are relatively low (hunters use their own smartphones), so the main cost is the software and database. The final property to consider is *circumvention*. Following the evaluation, several areas were identified where potential presentation or other forms of attack could occur. Addressing these vulnerabilities represents an important challenge for future development. Another key direction for future research is to extend the testing and validation of the method to additional wild ungulate species to assess its precision and general applicability.

## Data Availability

The raw data supporting the conclusions of this article will be made available by the authors, without undue reservation.
